# Cytotoxic drugs efficacy correlates with adipose tissue docosahexaenoic acid level in locally advanced breast carcinoma

**DOI:** 10.1038/sj.bjc.6690281

**Published:** 1999-04

**Authors:** P Bougnoux, E Germain, V Chajès, B Hubert, C Lhuillery, O Le Floch, G Body, G Calais

**Affiliations:** Laboratoire de Biologie des Tumeurs et Clinique d'Oncologie-Radiothérapie;, Service d'Information Médicale et d'Hygiène, Hôpital Bretonneau, Tours, 37044, France; LNSA, INRA, Jouy-en-Josas, 78350, France; Service de Gynécologie (UPRES-EA2103), Hôpital Bretonneay, Tours, 37044, France

**Keywords:** n-3 fatty acids, adipose tissue, docosahexaenoic acid, breast carcinoma, chemosensitivity

## Abstract

Experimental studies indicated that long-chain polyunsaturated fatty acids may increase sensitivity of mammary tumours to several cytotoxic drugs. To evaluate this hypothesis in breast cancer, we have prospectively studied the association between levels of fatty acids stored in breast adipose tissue and the response of the tumour to chemotherapy in 56 patients with an initially localized breast carcinoma. Adipose breast tissue was obtained at the time of biopsy, and individual fatty acids were measured as a percentage of total fatty acids using capillary gas chromatography. Patients then received primary chemotherapy, combining mitoxantrone, vindesine, cyclophosphamide and 5-fluorouracil every 4 weeks. Tumour size was reassessed after three cycles of chemotherapy. Tumour response was evaluated according to World Health Organization criteria. Complete or partial response to chemotherapy was achieved in 26 patients (47%). Level of n-3 polyunsaturated fatty acids in adipose tissue was higher in the group of patients with complete or partial response to chemotherapy than in patients with no response or with tumour progression (*P* < 0.004). Among n-3 polyunsaturated, only docosahexaenoic acid (22:6n-3) was significantly associated with tumour response (*P* < 0.005). In a logistic regression analysis taking into account age, body mass index and tumour size, 22:6 n-3 level proved to be an independent predictor for chemosensitivity (*P* = 0.03). These results suggest that, in breast cancer, 22:6 n-3 may increase the response of the tumour to the cytotoxic agents used. © 1999 Cancer Research Campaign


British Journal of Cancer (1999) **79**(11/12), 1765–1769
© 1999 Cancer Research Campaign
Article no. bjoc.1998.0281

### Cytotoxic drugs efficacy correlates with adipose tissue docosahexaenoic acid level in locally advanced breast carcinoma

**P Bougnoux[1], E Germain[1], V Chajès[1], B Hubert[2], C Lhuillery[3], O Le Floch[1], G Body[4] and G Calais[1]**

[1]Laboratoire de Biologie des Tumeurs et Clinique d'Oncologie-Radiothérapie;[2]Service d'Information Médicale et d'Hygiène, Hôpital Bretonneau, 37044 Tours; [3]LNSA, INRA; 78350 Jouy-en-Josas; [4]Service de Gynécologie (UPRES-EA2103), Hôpital Bretonneay, 37044 Tours, France

**Summary** Experimental studies indicated that long-chain polyunsaturated fatty acids may increase sensitivity of mammary tumours to several cytotoxic drugs. To evaluate this hypothesis in breast cancer, we have prospectively studied the association between levels of fatty acids stored in breast adipose tissue and the response of the tumour to chemotherapy in 56 patients with an initially localized breast carcinoma. Adipose breast tissue was obtained at the time of biopsy, and individual fatty acids were measured as a percentage of total fatty acids using capillary gas chromatography. Patients then received primary chemotherapy, combining mitoxantrone, vindesine, cyclophosphamide and 5-fluorouracil every 4 weeks. Tumour size was reassessed after three cycles of chemotherapy. Tumour response was evaluated according to World Health Organization criteria. Complete or partial response to chemotherapy was achieved in 26 patients (47%). Level of n-3 polyunsaturated fatty acids in adipose tissue was higher in the group of patients with complete or partial response to chemotherapy than in patients with no response or with tumour progression ($P < 0.004$). Among n-3 polyunsaturated, only docosahexaenoic acid (22:6n-3) was significantly associated with tumour response ($P < 0.005$). In a logistic regression analysis taking into account age, body mass index and tumour size, 22:6 n-3 level proved to be an independent predictor for chemosensitivity ($P = 0.03$). These results suggest that, in breast cancer, 22:6 n-3 may increase the response of the tumour to the cytotoxic agents used.

**Keywords:** n-3 fatty acids; adipose tissue; docosahexaenoic acid; breast carcinoma; chemosensitivity

Breast cancer is the most common malignancy among women in the Western world. In many instances, breast cancer at diagnosis is a systemic disease that consequently requires adjuvant therapy with hormones or cytotoxic drugs. One important determinant of breast cancer growth and development is tumour cell sensitivity to cytotoxic agents that are given during initial treatment, either as induction chemotherapy or as adjuvant treatment to locoregional therapy. The occurrence of resistance of tumours to chemotherapy is one of the main obstacles to the successful chemotherapeutic treatment of cancer. Thus, factors which may increase sensitivity of tumours to anti-cancer drugs would increase survival of patients.

Experimental studies indicated that several fatty acids may modulate the sensitivity of tumour cells to several cytotoxic drugs. In vitro studies showed that sensitivity of mammary tumour cells to the cytotoxic drugs doxorubicin and mitoxantrone was increased by some polyunsaturated fatty acids (PUFA) supplemented in the medium and/or integrated into membrane phospholipids (Burns and Spector, 1994). Among long-chain PUFA, fatty acids of the n-3 series, and specifically docosahexaenoic acid (DHA, 22:6n-3), have been reported to increase the cytotoxic efficacy of doxorubicin on L1210 murine leukaemia cells (Guffy et al, 1984), on small-cell lung carcinoma cell lines (Zijlstra et al, 1987), and on a breast cancer cell line (Germain et al, 1998). Besides cell cultures, dietary fatty acids also influenced sensitivity of mammary tumours to several cytotoxic drugs in experimental animals. In a model of transplantable human mammary carcinoma in athymic mice, dietary fish oil enriched in n-3 PUFA has been found to enhance efficacy of anti-cancer drugs doxorubicin (Borgeson et al, 1989), cyclophosphamide (Shao et al, 1997) and mitomycin C (Borgeson et al, 1989; Shao et al, 1995).

Although experimental observations suggested that PUFA, and particularly n-3 PUFA, increased the sensitivity of mammary tumour cells to several anti-cancer drugs, no data in humans is available concerning the potential influence of dietary fatty acids on the chemosensitivity of tumours to cytotoxic drugs. However, circumstantial evidence suggests that a phenomenon similar to those revealed in experimental studies may operate in humans. In breast cancer patients who had undergone treatment, estimated dietary total fat and saturated fat were associated with risk for treatment failure in a subpopulation of patients with oestrogen receptor-rich tumours, suggesting that dietary fatty acids may affect the response of breast cancer to treatment (Holm et al, 1993). In a previous study, we found that a decreased level of alpha-linolenic acid (essential fatty acid of the n-3 family, 18:3 n-3) in adipose tissue of breast cancer patients was associated with subsequent development of visceral metastases (Bougnoux et al, 1994). These data suggest that the type of fatty acids available to tumour cells may have influenced the outcome of breast cancer by altering the response of cancer cells to the initial treatment.

To obtain direct evidence of an association between PUFA and tumour response to chemotherapy, we have prospectively examined the association between the level of individual fatty acids in

*Received 4 March 1998*
*Revised 17 July 1998*
*Accepted 30 July 1998*

*Correspondence to:* P Bougnoux, CORAD, Hôpital Bretonneau, F-37044 Tours, France the adipose breast tissue, used as an indicator of stored fatty acids, and the response of the breast tumour to primary (neoadjuvant) chemotherapy in 56 patients with locally advanced breast carcinoma. We found that a high level of docosahexaenoic acid (22:6 n-3, DHA) in breast adipose tissue was related to the response of the primary tumour to neoadjuvant chemotherapy, whereas a low level of DHA was associated to no response or even to tumour growth. This observation suggests that n-3 fatty acids available to the tumour may modulate in vivo the response of the tumour to the cytotoxic agents used.

### SUBJECTS AND METHODS

### Characteristics of patients and disease

Fifty-six patients with histologically confirmed, non-metastatic invasive breast carcinoma were entered into the study between September 1987 and February 1992 when the following criteria were met: a specimen of adipose tissue had been obtained during surgery; pathology, staging (TNM) and treatment had been performed at the University Hospital of Tours; and follow-up had been expected to be possible. Mean age of patients was 51.7 years (range 25–69). Distribution of patients according to age, menopause and body mass index (BMI) is presented in Table 1.

All patients had their medical history recorded and complete clinical examination by a gynaecological surgeon and a radiation oncologist. Chest radiograph, liver and cardiac ultrasound examination, isotope bone scan, as well as any additional investigation required were performed to assess the extent of disease. All patients had basal biological assessment (haematology, liver and kidneys, and serum tumour markers).

Tumour size was evaluated before surgery by clinical examination and mammographic assessment of tumour diameters, according to the World Health Organization (WHO) recommendations (Miller et al, 1981). Mean tumour size was determined by measuring the product of the two largest diameters (Tonkin et al, 1985). Surgical biopsy was performed before chemotherapy for pathological diagnosis and tissue markers assays.

The pathological characteristics of the tumours were reviewed by a single pathologist. Histopathological types were defined according to the WHO classification (Types histologiques des tumeurs du sein, Organisation Mondiale de la Santé; classification histologique internationale des tumeurs no. 2, 1981, Genève). All tumours were carcinoma, with 44 tumours belonging to the ductal type, six to the lobular, and six tumours to undetermined or to other types. Histoprognostic grading (SBR grade) was based on the evaluation of mitosis, tubular formation and nuclear pleomorphism, according to Bloom and Richardson (1957). Vascular invasion was defined by presence of tumour cells within lumen of blood or lymph vessels. Oestrogen and progesterone receptors were measured in tumour cytosol with an immunoenzymoassay (Abbott, USA).

### Primary chemotherapy

The combination of drugs used as primary chemotherapy was mitoxantrone (12 mg $m^{-2}$) by bolus injection (b.i.) on day 1 and on days 1 and 8, an administration of vindesine (2 mg $m^{-2}$) (b.i.), cyclophosphamide (500 mg $m^{-2}$) and 5-fluorouracile (700 mg $m^{-2}$) by short infusion. The cycle was repeated starting on day 29. Nine first patients were given a regimen in which epirubicin, 25 mg $m^{-2}$ (b.i.) on days 1 and 8, replaced mitoxantrone. All patients completed three cycles within the time frame scheduled. There was no acute toxicity of grade greater than 3 according to WHO grading. No patients received hormonal therapy simultaneously with primary chemotherapy.

### Anti-tumour end point of chemotherapy

To evaluate the chemosensitivity of tumours, tumour response was assessed after completion of three cycles of primary chemotherapy by both a surgical and a medical oncologist on the basis of the reduction in the product of the two largest diameters obtained through clinical and mammographic reassessment. Complete response (CR) was defined by complete disappearance of all evidence of tumour. Partial response (PR) was defined by at least 50% reduction in the products of largest diameters. Stable disease (SD) was defined as less than 50% reduction but no more than 25% increase in the products of largest diameters, and progressive disease (PD) as greater than 25% increase. When tumour regression was 50% or less, a mastectomy was performed, followed by radiation therapy using modalities reported elsewhere (Calais et al, 1994) and by six additional cycles of chemotherapy. When regression was greater than 50%, local treatment was radiation alone by external radiotherapy and interstitial implant as a boost to the initial location of the tumour, followed by six additional cycles of chemotherapy.

### Adipose tissue preparation and fatty acids analysis

Adipose tissue was obtained during biopsy, freed from epithelial breast or carcinoma tissue, washed in saline and kept frozen in liquid nitrogen until analysis. Laboratory analyses were blinded on the links between samples and disease status of subjects (tumour response). The procedures for preparation of fatty acids have been reported elsewhere (Bougnoux et al, 1994). In summary, total lipids of the adipose tissue were extracted and triglycerides purified on silica gel G thin-layer chromatographic plates. Fatty acids were analysed as methyl esters by gas chromatography on a fused-silica capillary column with a liquid phase of carbowax 20 M, using an on-column injector and a flame ionization detector. Fatty acids were identified and quantified with the use of commercial standards (Nu-Check-Prep, Elisian, MN, USA). Fatty acids were expressed as percentage of total fatty acids area. Unidentified peaks amounted for less than 3%. All solvents were high-performance liquid chromatography (HPLC) grade, and nitrogen was used at each step to protect PUFA from peroxidation. Intra- and interassay coefficients of variation (CV) have been already reported in detail (Chajès et al, 1992). CV were less than 1% for large peaks and reached 10% for the smallest peaks.

### Statistical analysis

Data were analysed using the EPI-INFO and BMDP statistical software. Associations between fatty acids taken either individually or as nutritional classes and clinical characteristics of the patients were assessed by the Kruskall–Wallis non-parametric test. All variables associated with response to chemotherapy were assessed by unconditional logistic regression with BMDP software (Dixon, 1985).

 © Cancer Research Campaign 1999

**Table 1** Clinical characteristics of patients and breast tumours and response to chemotherapy

| Clinical factor | Number of patients | Objective response[a] to chemotherapy (%) | *P*-value[b] |
|---|---|---|---|
| Age | | | |
| < 50 | 26 | 35 | |
| ≥ 50 | 30 | 57 | NS[c] |
| Menopausal status | | | |
| Premenopausal | 30 | 47 | |
| Post-menopausal | 26 | 50 | NS |
| Body mass index | | | |
| < 25 | 26 | 39 | |
| ≥25 | 30 | 53 | NS |
| Tumour clinical size | | | |
| ≤ 50 mm | 25 | 60 | |
| > 50 mm | 31 | 36 | 0.06 |
| Tumour mammographic size | | | |
| ≤ 50 mm | 41 | 54 | |
| > 50 mm | 15 | 27 | 0.07 |
| Nodal status[d] | | | |
| Negative | 24 | 54 | |
| Positive | 32 | 41 | NS |
| Histoprognostic grade[e] | | | |
| I or II | 35 | 43 | |
| III | 21 | 52 | NS |
| Vascular invasion[f] | | | |
| Absent | 32 | 41 | |
| Present | 22 | 50 | NS |
| Oestrogen receptor | | | |
| > 10 fmol $mg^{-1}$ | 45 | 47 | |
| ≤ 10 fmol $mg^{-1}$ | 11 | 46 | NS |
| Progesterone receptor | | | |
| > 10 fmol $mg^{-1}$ | 37 | 54 | |
| ≤ 10 fmol $mg^{-1}$ | 19 | 32 | NS |

[a]CR or PR (>50%). [b]Chi-squared test. [c]Not significant. [d]Clinical staging. [e]Scarff and Bloom. [f]Unavailable for two patients.

### RESULTS

### Description of patients

Among the 56 patients studied, 26 (47%) displayed a complete (five) or partial (21) response to chemotherapy, whereas progressive disease occurred in one. Chemosensitivity was not significantly associated with any clinical factors, although a trend was observed for an inverse association of tumour size (clinical or mammographic) to response of tumours to chemotherapy (Table 1).

### Relationship between adipose tissue fatty acid levels and tumour chemosensitivity

Mean levels of individual fatty acids were compared according to tumour response, or not, to chemotherapy (Table 2). Levels of total n-3 PUFA taken as a family of polyunsaturated fatty acids was higher in the group of patients with an objective (CR or PR > 50%) response to chemotherapy than in patients with no response (SD or PD) ($P$ = 0.004). Among n-3 PUFA, only an elevated 22:6n-3 level was significantly associated with an objective response to chemotherapy ($P$ = 0.005). Breast adipose tissue levels of total saturates, monounsaturates, or n-6 PUFA were similar between patients with a response and patients with no response to chemotherapy.

### Relationship between adipose tissue fatty acid levels and characteristics of patients and disease

Levels of total n-3 PUFA, or levels of 22:6 n-3 in adipose breast tissue, were found to be significantly higher in patients ≥ 50 years old ($P < 0.001$, $P < 0.01$ respectively) or in post-menopause ($P < 0.01$, $P < 0.05$ respectively). Level of 22:6n-3 was also higher in patients with BMI ≥ 25.0 ($P < 0.05$). No significant association was found between n-3 PUFA and any of the other clinical factors (data not shown).

### Multivariate analysis

To control for these possible confounding factors, a logistic regression analysis was performed. The following continuous variables were entered into the models: age, BMI, mammographic size, adipose tissue level of n-3 PUFA or 22:6n-3.

The 22:6n-3 level proved to be an independent predictor for response to chemotherapy ($P$ = 0.03, likelihood ratio test). When 22:6n-3 was categorized into two classes, using the median value (0.15%) as a threshold, we found that the risk of tumour response to chemotherapy was multiplied by 4.6 when the 22:6n-3 level was above the median value (Table 3). When the n-3 PUFA level was adjusted for the three potential confounding factors, the association between this group of fatty acids and tumour response to chemotherapy did not reach a significant level; the risk of response to chemotherapy was 3.2 times higher when the n-3 PUFA level was above the median value (0.78%), used as a threshold, compared with when the level was below the median value (Table 3).

### DISCUSSION

The purpose of this study was to determine whether the response of breast carcinoma to anti-cancer drugs might be influenced by the type of fatty acids available to the tumour. To examine this hypothesis, we have prospectively studied the association between the level of individual fatty acids in the adipose breast tissue, used as an indicator of stored fatty acids, and the response of the tumour to primary (neoadjuvant) chemotherapy in 56 patients with localized breast carcinoma. Breast cancer patients presenting with operable breast tumour larger than 3 cm and undergoing primary chemotherapy as the first step in their treatment represent an appropriate and simple system for evaluating the influence of host parameters on chemosensitivity of their tumour because tumour size is readily measurable and adipose tissue contiguous to the tumour is readily available during diagnosis procedures. We found an association of elevated DHA level in the adipose breast tissue with chemosensitivity of the breast tumour. We observed that the degree of tumour shrinkage after primary chemotherapy was highest in patients with elevated levels of DHA in breast adipose tissue. Although n-3 PUFA content of adipose tissue increased with age, menopause and BMI, DHA proved to be an independent predictive factor of tumour chemosensitivity. This predictive value of adipose breast tissue DHA levels on tumour chemosensitivity is unique because no host-dependent parameter has been available for predicting the probability of tumour response to anti-cancer agents. These data suggest that n-3 PUFA available to the tumour may modulate the tumour response to chemotherapy in humans.

© Cancer Research Campaign 1999

**Table 2** Fatty acids in adipose breast tissue according to tumour response to primary chemotherapy

| **Fatty acid (%)**[a] | **No response (SD + PD) (*n* = 30) Mean** | **Objective response (CR + PR > 50%) (*n* = 26) Mean** | ***P*-value**[b] |
|---|---|---|---|
| Saturates | | | |
| 16:0 (palmitic acid) | 23.27 | 22.76 | NS[c] |
| 18:0 (stearic acid) | 5.78 | 5.52 | NS |
| Total saturates[d] | 32.95 | 32.08 | NS |
| Monounsaturates | | | |
| 16:1 (palmitoleic acid) | 3.75 | 3.76 | NS |
| 18:1 (oleic acid) | 42.45 | 42.24 | NS |
| Total monounsaturates[e] | 47.44 | 47.40 | NS |
| Polyunsaturates n-6 | | | |
| 18:2 n-6 (linoleic acid) | 14.90 | 15.24 | NS |
| 20: 4 n-6 (arachidonic acid) | 0.33 | 0.40 | 0.07 |
| 22:4 n-6 (adrenic acid) | 0.18 | 0.22 | NS |
| Total n-6[f] | 15.92 | 16.39 | NS |
| Polyunsaturates n-3 | | | |
| 18:3 n-3 ($\alpha$-linolenic acid) | 0.38 | 0.44 | NS |
| 22:5 n-3 (docosapentaenoic acid) | 0.21 | 0.26 | 0.06 |
| 22:6 n-3 (docosahexaenoic acid, DHA) | 0.14 | 0.20 | 0.005 |
| Total n-3 | 0.73 | 0.90 | 0.004 |

[a]Percentage of total fatty acids. [b]Kruskal–Wallis test. [c]Not significant ($P \geq 0.10$). [d]Included: 14:0, 15:0, 17:0, 20:0, 22:0, 24:0. [e]Included: 14:1, 20:1, 22:1. [f]Included: 18:3 n-6, 20:2 n-6, 20:3 n-6.

**Table 3** Predictive value of docosahexaenoic acid on tumour response to chemotherapy, adjusted for age, body mass index and tumour size

| **Variable** | **Adjusted odds ratio**[a] | **95% CI** | ***P*-value**[b] |
|---|---|---|---|
| Docosahexaenoic acid (22:6 n-3) | | | |
| < 0.15% | 1 | | |
| >0.15% | 4.6 | 1.2–18.5 | 0.03 |
| Polyunsaturated fatty acids (n-3 PUFA) | | | |
| < 0.87% | 1 | | |
| > 0.87% | 3.2 | 0.8–12.6 | 0.10 |

[a]Unconditional logistic regression. [b]Likelihood ratio test; %, median value.

Tumour mass results from several components involved in tumour growth. These include tumour cell proliferation, as well as tumour cell loss (Steel, 1979). Experimental data from animal studies suggest that dietary n-3 PUFA (originating from marine oils) inhibit mammary tumour growth by increasing tumour cell loss (Gabor and Abraham, 1986; Gonzalez et al, 1991, 1993). In our study, no link was observed between the mitotic index found in the carcinoma and any individual fatty acid measured in the adipose tissue (data not shown). Therefore, the association found between elevated long-chain n-3 PUFA and reduction in the size of the tumour, after chemotherapy, suggests that these fatty acids may increase the tumour cell loss component of the tumour rather than decrease cell proliferation in response to chemotherapy.

The association of chemosensitivity of breast carcinoma with an elevated level of n-3 PUFA, particularly DHA, in adipose breast tissue can be interpreted in different ways. The first possible interpretation is that n-3 PUFA available to the tumour influenced chemosensitivity through an alteration of the physical state of tumour cell membranes. We previously reported that membrane fatty acids of breast carcinoma result from intrinsic properties of the tumour and also from host fatty acids available to the tumour (Chajès et al, 1995*a*). Therefore, if we assume that patients with elevated levels of DHA in their adipose breast tissue have elevated levels of this fatty acid in membrane phospholipids of their breast tumour, this biochemical change would result in an increase in tumour cell membrane fluidity, an event which has been linked with chemosensitivity (Siegfried et al, 1983; Burns and Spector, 1994). Putative mechanisms due to an increase in membrane fluidity of tumour cells may involve an increased passive diffusion of cytotoxic drugs (Pelletier et al, 1990), resulting in an intracellular accumulation of drugs (Burns et al, 1998; Callaghan et al, 1993).

An alternative interpretation is that n-3 PUFA available to the tumour may modulate chemosensitivity of breast carcinoma through an increased formation of peroxides in the tumour. In mammary tumour cells in vitro (Chajès et al, 1995*b*) or in human breast cancer cells growing in nude mice (Gonzalez et al, 1991, 1993), supplementation of medium or diet with n-3 PUFA led to an increase in lipid peroxidation products in tumour cells and to a suppression of breast cancer growth, suggesting that PUFA may interfere with tumour cell growth through lipid peroxides formation. In human mammary carcinoma grafted to nude mice, the activities of some enzymes involved in the oxidative stress were increased by feeding high levels of corn oil, enriched in n-6 PUFA (Shao et al, 1994), or menhaden oil, enriched in n-3 PUFA (Shao et al, 1995), resulting in an enhanced antineoplastic effect of the drug mitomycin C. Some experimental studies evaluated whether lipid peroxidation products from PUFA in tumour cells would correlate with the sensitivity of tumour cells to cytotoxic drugs. In mammary tumour cells in vitro, we found that various n-6 or n-3 PUFA combined with a pro-oxidant mixture (sodium ascorbate/2-methyl-1,4-naphthoquinone) increased both cytotoxic efficacy of the anti-cancer drug doxorubicin and level of peroxides in tumour cells, with the strongest effects obtained with DHA (Germain et al,

*© Cancer Research Campaign 1999*

1998). In a similar way, in implanted human breast tumours in mice, the use of fish oil and pro-oxidant iron supplemented with the drug edelfosine resulted in an increase in both the level of lipid peroxidation products in tumours and the cytotoxic efficacy of the drug (Hardman et al, 1997). In our study, we found that, among all PUFAs measured in adipose tissue, DHA, the most unsaturated fatty acid and therefore a good substrate for peroxidation, was related to the highest tumour shrinkage subsequent to the action of cytotoxic drugs. This observation may suggest that a higher level of DHA in adipose tissue could increase the cytotoxic efficacy of drugs on breast carcinoma by providing a higher availability in the substrate for peroxides formation.

The exact mechanism by which lipid peroxides may modulate tumour sensitivity to cytotoxic drugs remains to be elucidated. Free radicals and peroxides formed during lipid peroxidation may physically damage cellular proteins, DNA (Masotti et al, 1988) and lipid membranes including those of mitochondria, which are a key target for chemotherapy-induced apoptosis (Decaudin et al, 1998).

Insights gained into mechanisms involved in the sensitivity of tumours to anti-cancer agents are likely to lead to new strategies in the treatment of cancer. If certain fatty acids available to the tumour, particularly 22:6n-3, appear to increase the response of tumours to chemotherapy, the next question is which factors might influence DHA levels in breast adipose tissue in breast cancer patients. DHA levels in adipose tissue have been shown to reflect long-term dietary intake of estimated n-3 PUFA or fish intake (London et al, 1991), and an enrichment in the diet of breast cancer patients with n-3 PUFA led to increased levels of DHA in breast adipose tissue (Bagga et al, 1997). Thus, dietary intervention could provide an effective means of increasing DHA availability in tumour tissues and thereby may affect chemosensitivity of tumours. We are currently investigating the mode of action of dietary n-3 fatty acids on the chemosensitivity of mammary tumours in a rodent experimental system.

### ACKNOWLEDGEMENTS

We thank F Fetissof for the pathology, A Reynaud-Bougnoux, S Chapet, C Berger and A Fignon, S Avigdor and J Lansac for the careful appraisal of tumour response. This work was supported in part by grants from INSERM (C.R.E. no. 930301) and from the Ligue Nationale Contre le Cancer (Comités du Cher, de la Charente et du Loir-et-Cher).

### REFERENCES

Bagga D, Capone S, Wang HJ, Heber, Lill M, Chap L and Glaspy JA (1997) Dietary modulation of omega-3/omega-6 polyunsaturated fatty acid ratios in patients with breast cancer. *J Natl Cancer Inst* **89**: 1123–1131

Bloom HJG and Richardson WW (1957) Histologic grading and prognosis in breast cancer. *Br J Cancer* **11**: 359–377

Borgeson CE, Pardini L, Pardini RS and Reitz R (1989) Effects of dietary fish oil on human mammary carcinoma and on lipid-metabolizing enzymes. *Lipids* **24**: 290–295

Bougnoux P, Koscielny S, Chajès V, Descamps P, Couet C and Calais G (1994) Alpha-linolenic acid content of adipose tissue: a host determinant of the risk of early metastasis in breast cancer patients. *Br J Cancer* **70**: 330–334

Burns CP and Spector AA (1994) Biochemical effects of lipids on cancer therapy. *J Nutr Biochem* **5**: 114–123

Burns CP, Haugstad BN, Mossman CJ, North JA and Ingraham LM (1988) Membrane lipid alteration: effect on cellular uptake of mitoxantrone. *Lipids* **23**: 393–397

Calais G, Berger C, Descamps P, Chapet S, Reynaud-Bougnoux A, Body G, Bougnoux P, Lansac J and Le Floch O (1994) Conservative treatment feasibility with induction chemotherapy, surgery, and radiotherapy for patients with breast carcinoma larger than 3 cm. *Cancer* **74**: 1283–1288

Callaghan R, Stafford A and Epand M (1993) Increased accumulation of drugs in multidrug resistant cell line by alteration of membrane biophysical properties. *Biochim Biophys Acta* **1175**: 277–282

Chajès V, Niyongabo T, Lanson M, Fignon A, Couet C and Bougnoux P (1992) Fatty-acid composition of breast and iliac adipose tissue in breast-cancer patients. *Int J Cancer* **50**: 405–408

Chajès V, Lanson M, Fetissof F, Lhuillery C and Bougnoux P (1995*a*) Membrane fatty acids of breast carcinoma: contribution of host fatty acids and tumor properties. *Int J Cancer* **63**: 169–175

Chajès V, Sattler W, Stranzl A and Kostner GM (1995*b*) Influence of n-3 fatty acids on the growth of human breast cancer cells in vitro: relationship to peroxides and vitamin E. *Breast Cancer Res Treat* **34**: 199–212

Decaudin D, Maezo I, Brenner C and Kroemer G (1998) Mitochondria in chemotherapy-induced apoptosis: a prospective novel target of cancer therapy. *Int J Oncol* **12**: 141–152

Dixon DJ (1985) *BMDP Statistical Software* Berkeley, CA: University of California Press

Gabor H and Abraham S (1986) Effect of dietary menhaden oil on tumor cell loss and the accumulation of mass of a transplantable mammary adenocarcinoma in BALBc mice. *J Natl Cancer Inst* **76**: 1223–1229

Germain E, Chajès V, Cognault S, Lhuillery C and Bougnoux P (1998) Enhancement of doxorubicin cytotoxicity by polyunsaturated fatty acids in the human breast tumor cell line MDA-MB-231: relationship to lipid peroxidation. *Int J Cancer* **75**: 578–583

Gonzalez MJ, Schemmel RA, Gray JI, Dugan L, Sheffield LF and Welsch CW (1991) Effect of dietary fat on growth of MCF-7 and MDA-MB231 human breast carcinomas in athymic mice: relationship between carcinoma growth and lipid peroxydation product levels. *Carcinogenesis* **12**: 1231–1235

Gonzalez MJ, Schemmel RA, Dugan L, Gray JI and Welsch CW (1993) Dietary fish oil inhibits human breast carcinoma growth: a function of increased lipid peroxidation. *Lipids* **28**: 827–832

Guffy MM, North JA and Burns CP (1984) Effect of cellular fatty acid alteration on adriamycin sensitivity in cultured L1210 murine leukemia cells. *Cancer Res* **44**: 1863–1866

Hardman WE, Barnes CJ, Knight CW and Cameron IL (1997) Effects of iron supplementation and ET-18-OCH3 on MDA-MB-231 breast carcinomas in nude mice consuming a fish oil diet. *Br J Cancer* **76**: 347–354

Holm LE, Nordevang E, Hjalmar ML, Lidbrink E, Callmer E and Nilsson B (1993) Treatment failure and dietary habits in women with breast cancer. *J Natl Cancer Inst* **85**: 32–36

London SJ, Sacks FM, Caesar J, Stampfer MJ, Siguel E and Willett WC (1991) Fatty acid composition of subcutaneous adipose tissue and diet in postmenopausal US women. *Am J Clin Nutr* **54**: 340–345

Masotti L, Casali E and Galeotti T (1988) Lipid peroxidation in tumor cells. *Free Radical Biol Med* **4**: 377–386

Miller AB, Hoogstraten B, Staquet M and Winkler A (1981) Reporting results of cancer treatment. *Cancer* **47**: 207–214

Pelletier H, Millot JM, Chauffert B, Manfait M, Genne P and Martin F (1990) Mechanisms of resistance of confluent human and rat colon cancer cells to anthracyclines: alteration of drug passive diffusion. *Cancer Res* **50**: 6626–6631

Shao Y, Pardini L and Pardini RS (1994) Enhancement of the antineoplastic effect of mitomycin C by dietary fat. *Cancer Res* **54**: 6452–6457

Shao Y, Pardini L and Pardini RS (1995) Dietary menhaden oil enhances mitomycin C antitumor activity toward human mammary carcinoma MX-1. *Lipids* **30**: 1035–1045

Shao Y, Pardini L and Pardini RS (1997) Intervention of transplantable human mammary carcinoma MX-1 chemotherapy with dietary menhaden oil in athymic mice: increased therapeutic effects and decreased toxicity of cyclophosphamide. *Nutr Cancer* **28**: 63–73

Siegfried JA, Kennedy KA, Sartorelli AC and Tritton TR (1983) The role of membranes in the mechanism of action of the antineoplastic adriamycin. *J Biol Chem* **258**: 339–343

Steel GG (1979) Terminology in the description of drug-radiation interactions. *Int J Radiat Oncol Biol Phys* **5**: 1145–1150

Tonkin K, Tritchler D and Tannock I (1985) Criteria of tumor response in clinical trials of chemotherapy. *J Clin Oncol* **3**: 870–875

Zijlstra JG, de Vries EGE, Muskriet FAJ, Martini IA, Timmer-Bosscha H and Mulder NH (1987) Influence of docosahexaenoic acid *in vitro* on intracellular adriamycin concentration in lymphocytes and human adriamycin sensitive and resistant small-cell lung cell lines, and on cytotoxicity in the tumor cell lines. *Int J Cancer* **90**: 850–856

© Cancer Research Campaign 1999
